# Extracellular α-Synuclein Leads to Microtubule Destabilization *via* GSK-3β-Dependent Tau Phosphorylation in PC12 Cells

**DOI:** 10.1371/journal.pone.0094259

**Published:** 2014-04-10

**Authors:** Magdalena Gąssowska, Grzegorz A. Czapski, Beata Pająk, Magdalena Cieślik, Anna M. Lenkiewicz, Agata Adamczyk

**Affiliations:** 1 Department of Cellular Signalling, Mossakowski Medical Research Centre Polish Academy of Sciences, Warsaw, Poland; 2 Electron Microscopy Platform, Mossakowski Medical Research Centre Polish Academy of Sciences, Warsaw, Poland; McGill University Department of Neurology and Neurosurgery, Canada

## Abstract

α-Synuclein (ASN) plays an important role in pathogenesis of Parkinson's disease (PD) and other neurodegenerative disorders. Novel and most interesting data showed elevated tauopathy in PD and suggested relationship between ASN and Tau protein. However, the mechanism of ASN-evoked Tau protein modification is not fully elucidated. In this study we investigated the role of extracellular ASN in Tau hyperphosphorylation in rat pheochromocytoma (PC12) cells and the involvement of glycogen synthase kinase-3β (GSK-3β) and cyclin-dependent kinase 5 (CDK5) in ASN-dependent Tau modification. Our results indicated that exogenously added ASN increases Tau phosphorylation at Ser396. Accordingly, the GSK-3β inhibitor (SB-216763) prevented ASN-evoked Tau hyperphosphorylation, but the CDK5 inhibitor had no effect. Moreover, western blot analysis showed that ASN affected GSK-3β via increasing of protein level and activation of this enzyme. GSK-3β activity evaluated by its phosphorylation status assay showed that ASN significantly increased the phosphorylation of this enzyme at Tyr216 with parallel decrease in phosphorylation at Ser9, indicative of stimulation of GSK-3β activity. Moreover, the effect of ASN on microtubule (MT) destabilization and cell death with simultaneous the involvement of GSK-3β in these processes were analyzed. ASN treatment increased the amount of free tubulin and concomitantly reduced the amount of polymerized tubulin and SB-216763 suppressed these ASN-induced changes in tubulin, indicating that GSK-3β is involved in ASN-evoked MT destabilization. ASN-induced apoptotic processes lead to decrease in PC12 cells viability and SB-216763 protected those cells against ASN-evoked cytotoxicity. Concluding, extracellular ASN is involved in GSK-3β-dependent Tau hyperphosphorylation, which leads to microtubule destabilization. GSK-3β inhibition may be an effective strategy for protecting against ASN-induced cytotoxicity.

## Introduction

α-synuclein (ASN) is a 140-amino acid, presynaptic protein which in physiological conditions is involved in dopaminergic system function [1], [2]. However, under pathological conditions ASN changes its native conformation, becomes an insoluble self-aggregate to form oligomers and polymers and accumulates into intraneuronal inclusions called Lewy bodies (LBs) [3], [4]. ASN in its oligomeric form plays a key role in the pathogenesis of Parkinson's disease (PD) and other synucleinopathies, but the mechanism by which ASN contributes to neural degeneration remains unknown [5]. More recent evidence suggests that extracellular ASN oligomers play a pivotal role in neurodegeneration progression [6], [7]. Also, our previous *in vitro* studies showed that ASN is secreted from the synaptic endings into the extracellular space during oxidative stress, and exogenously added ASN may evoke cell death [8].

A growing body of evidence suggests that ASN in neurodegenerative processes acts together with other amyloidogenic peptides, including amyloid-beta peptide (Aβ). In our previous studies we indicated that extracellularly applied ASN increases the secretion of Aβ and potentiates its toxicity, thus leading to mitochondrial dysfunction and caspase-dependent PC12 cell death [9]. Previously published data indicated a relationship between ASN and the microtubule associated protein (MAP) - Tau in neurodegenerative processes. Also, more often clinical evidence has shown a strong association between tauopathies and synucleinopathies, where abnormalities in both proteins, MAP-Tau and ASN, have been described [4], [10]–[13]. ASN-positive structures have been found in various brain regions in patients with sporadic and familial Alzheimer's disease (AD); LBs were detected in the amygdala of AD and Down's syndrome [4], [10], [11], [13]. Furthermore, in individuals with sporadic PD or dementia with LBs, Tau-immunoreactive LBs were observed [11].

The major function of Tau, like other MAPs, is stabilization and regulation of microtubule (MT) dynamics necessary for neurite outgrowth, morphogenesis, axonal transport and normal neuronal functions [14]. Therefore, the microtubule destabilization could cause neurodegeneration. Recently, it has been proposed that microtubule destabilization plays a role in dopaminergic neurons loss [15]. Tau can interact with the plasma membrane and may play a role in relaying signals to the cytoskeleton from the cell surface or the scaffolding signalling complexes [16]. Tau activity is regulated by phosphorylation/dephosphorylation cycles. Phosphorylation at specific sites detaches Tau from MTs and allows MT depolymerization, while Tau dephosphorylation enables it to bind and stabilize the MT [17]–[19]. Although Tau contains approximately 85 potential phosphorylation sites in its longest isoform, phosphorylation at Ser396 seems to play a pivotal role for its function and in particular destabilizes microtubules [19]–[22]. Increased Tau phosphorylation at Ser396 has been found in synaptic-enriched fractions both in AD frontal cortex and PD brains along with phosphorylated ASN [12], [19]. In neurodegenerative disorders, such as AD and PD, hyperphosphorylation of Tau leads to intracellular accumulation of this protein and the formation of neurofibrillary tangles (NFTs). In PD, Tau has been described as a component incorporated into the filaments in LBs and co-localized with ASN [18], [19]. It was suggested that toxic interaction with ASN may lead to hyperphosphorylation of Tau and, eventually, to the deposition of both proteins in PD [19].

The significance of extracellular ASN in Tau phosphorylation and MT instability, which could be involved in the mechanisms of dopaminergic cell death, is unknown. Therefore, this study was performed to investigate the effect of exogenous ASN on phosphorylation state of Tau, microtubule stability and death in PC12 cells. Moreover, the involvement of two major Tau-kinases: glycogen synthase kinase-3β (GSK-3β) and cyclin-dependent kinase (CDK5) in ASN-evoked Tau modification, MT destabilization and death was evaluated. Taken together, our data indicated that extracellular ASN induces GSK-3β-dependent Tau modulation and we suggest that proapoptotic effect of ASN might be mediated at least in part by the GSK-3β-catalyzed Tau hyperphosphorylation, which leads to the tubulin depolymerization and in further consequence to impairment of cytoskeleton stability. Finally, the effects of ASN can be largely prevented by inhibition of GSK-3β kinase. GSK-3β inhibition may be an effective strategy for protecting against ASN-induced cytotoxicity. Thus, one of the mechanisms by which ASN could mediate PC12 cell death is the disruption of tubulin polymerization into microtubules (microtubules destabilization), the cell's structural support system.

## Materials and Methods

### Reagents

The following antibodies were used in the current study: anti-phospho Tau (Ser396), anti-phospho GSK-3β (Ser9), anti-GSK-3β, anti-α/β-tubulin (Cell Signalling, Beverly, MA, USA), anti-phospho-GSK-3β (Ty216) (BD Biosciences Pharmingen, NJ, Franklin Lakes, USA), anti-GAPDH, anti-rabbit IgG (Sigma-Aldrich, St. Louis, MO, USA), anti-mouse IgG (GE Health Care UK, Little Chalfont, Buckinghamshire, UK), anti-mouse IgG conjugated with gold particles (Jackson Immunoresearch, West Grove, PA, USA). ASN was obtained from rPeptide (Bogart, GA, USA). Reagents for reverse transcription (High Capacity RNA-to-cDNA Master Mix) and PCR (Gene Expression Master Mix) were obtained from Applied Biosystems (Foster City, CA, USA). Cell culture reagents: Dulbecco's Modified Eagle's Medium (DMEM), Fetal Bovine Serum (FBS), Horse Serum (HS), penicillin, streptomycin, G418, L-glutamine, and other reagents such as: deoxyribonuclease I, 3-(4,5-dimethyl-2-tiazolilo)-2,5-diphenyl-2H-tetrazolium bromide (MTT), TRI-reagent, polyethylenoimine (PEI), dimethyl sulfoxide (DMSO), 2′-(4-hydroxyphenyl)-5-(4-methyl-1-piperazinyl)-2,5′-bi-1Hbenzimidazoletrihydrochloridehy

drate (Hoechst 33258), MES (2-N-(morpholino)ethanesulfonic acid), Colchicine and Paclitaxel (Taxol) were obtained from Sigma-Aldrich (St. Louis, MO, USA). Specific GSK-3 inhibitor: 3-(2,4-dichlorophenyl)-4-(1-methyl-1H-indol-3-yl)-1H-pyrrole-2,5-dione (SB-216763) was from Tocris Bioscience (Bristol, UK), potent inhibitor of CDK5: N-(5-Isopropylthiazol-2-yl)phenylacetamide) (BML-259) was from Enzo Life Sciences (Lausen, Switzerland). Cell Lysis Buffer (10×) was obtained from Cell Signalling (Beverly, MA, USA). Reagents for TUNEL assay: FITC-labelled dUTP and PI/RNase Staining Solution were obtained from BD Biosciences, Heidelberg, Germany.

### Preparation of soluble alpha-synuclein

Human alpha-synuclein (ASN, rPeptide, Bogart, GA, USA) was dissolved in phosphatebuffered saline (PBS) (pH 7.4) at a concentration of 100 μM and immediately used for experiments as soluble ASN in form of mixture of monomers and oligomers [23].

### Cell culture

The studies were carried out using rat pheochromocytoma (PC12) cells [24]–[26]. PC12 cells were a kind gift from Prof. Anne Eckert (Neurobiology Laboratory for Brain Aging and Mental Health University of Basel, Basel, Switzerland). Cells were cultured in DMEM supplemented with 10% heat-inactivated FBS, 5% heat-inactivated HS, 1% penicillin/streptomycin (50 U/ml) and 2 mM L-glutamine. Cells were maintained at 37°C in a humidified incubator in 5% CO_2_ atmosphere.

### Cell treatment protocols

Equal PC12 cell numbers were seeded into 96-well 0.1% PEI-coated plates or dishes and the growth medium was changed to a low-serum medium (DMEM supplemented with 2% FBS, 1% penicillin/streptomycin and 1% L-glutamine). Then PC12 cells were treated with exogenous ASN (10 μM), GSK-3 kinase inhibitor: SB-216763 (10 μM), CDK5 kinase inhibitor: BML-259 (10 μM), Colchicine (0.1 μM) and Taxol (0.1 μM) for 48 h.

### Determination of cell survival using MTT test – cytotoxicity assay

Cellular viability was evaluated by reduction of 2-(4,5-dimethylthiazol-2-yl)-2,5-diphenyltetrazolium bromide (MTT) to formazan. After 48 h treatment with the tested compounds, MTT (2.5 mg/ml) was added to all of the wells. Cells were incubated at 37°C for 2 h. Then medium was removed, the cells were dissolved in DMSO and absorbance at 595 nm was measured.

### Determination of apoptosis using Hoechst staining

The typical apoptotic nuclear morphology (nuclear condensation and shrinkage) was determined by microscopic analysis of the cells stained by Hoechst 33258. After incubation in the presence of ASN, cells were fixed by addition of 50 μl fresh mixture of methanol: glacial acetic acid (3∶1) for 3 min at RT to the culture medium. Cells were gently re-rinsed in methanol: glacial acetic acid (3∶1) for 5 min and left to dry for 30 min at RT. Next the cells were washed with PBS and stained with 1 μg/ml Hoechst 33342 (Sigma-Aldrich, St. Louis, MO, USA) for 30 min. To quantify the apoptotic process, cells with typical apoptotic nuclear morphology were examined under a fluorescence microscope (Olympus BX51, Japan). The results were expressed as the percentages of apoptotic cells.

### Determination of apoptosis using TUNEL assay

The presence of DNA fragmentation in apoptotic cells was determined by microscopic analysis of the cells stained by terminal deoxynucleotidyl transferase (TdT)-mediated dUTP- fluorescein-isothiocyanate (FITC)-nick end-labelling (TUNEL) method. After incubation in the presence of ASN, cells were fixed as described above (Hoechst staining). The TUNEL assay was performed according to Gavrieli et al. [27] with minor modifications. Shortly, fixed cells were incubated for 3 h at 37°C in a DNA labelling solution, containing TdT (15 U/well) and 40 pM FITC-labelled dUTP (BD Biosciences, Heidelberg, Germany). Incubation was terminated by repeated rinsing with TRIS buffer containing EDTA (10 mM TRIS, 1 mM EDTA) followed by extensive PBS washes. After the final wash, nuclei were stained with PI/RNase Staining Solution (BD Biosciences, Heidelberg, Germany). All fixed cells stain with PI, but only the later stage apoptotic cells show up as FITC positive. The FITC-TUNEL staining was visualized by fluorescence microscopy. The number of apoptotic (FITC-stained) cells and the total number (PI-stained) of cells were counted, and results were expressed as percentages of apoptotic cells.

### Determination of free and polymerized tubulin

The microtubules stability was analyzed according to method described by Hongo et al. [15]. Free and polymerized tubulins were extracted from PC12 cells using following protocol. The cells were washed twice with 1 ml of buffer A containing 0.1 M MES (pH 6.75), 1 mM MgSO_4_, 2 mM EGTA, 0.1 mM EDTA, and 4 M glicerol at 37°C. After incubating the cells at 37°C for 10 min in 400 μl of free tubulin extraction buffer (buffer A plus 0.1% Triton X-100 and protease inhibitors), the extracts were centrifuged at 37°C for 2 min at 16.000×g. The supernatant fractions contained free tubulin that had been extracted from the cytosol. The cell pellet and lysed cells on the bottom of tube were dissolved in 400 μl of 25 mM Tris (pH 6.8) with 0.5% SDS, which contained tubulin in its original polymerized state (i.e., microtubules). Equal amounts of total proteins from the free and polymerized tubulin fractions were analyzed by Western blot analysis using an anti-α/β-tubulin antibody.

### Post embedment immunostaining

Cells were fixed in 4% paraformaldehyde and 0.1% glutaraldehyde in 0.1 M PBS (pH 7.4) for 2 h at 4°C. After dehydration the cells were embedded in Epon 812, and ultrathin sections were processed according to the post-embedding procedure. Monoclonal mouse anti-phospho Tau (Ser396) was diluted 1∶30 in PBS and applied to the slices for 24 h at 4°C. Then the grids were exposed to secondary anti-mouse IgG conjugated with gold particles diluted 1∶50 in PBS. The sections were examined and photographed with a JEOL JEM-1011 electron microscope. Mean phospho-Tau expression in the control and ASN-treated cells was calculated as the number of gold particles in 10 randomly selected visual fields ± S.E.M.

### Real time polymerase chain reaction (RT-PCR)

Reverse transcription was performed using a High Capacity cDNA Reverse Transcription Kit according to the manufacturer's protocol (Applied Biosystems, Foster City, CA, USA). The level of mRNA for selected genes was analyzed by using TaqMan Gene Expression Assays (Applied Biosystems, Foster City, CA, USA) according to the manufacturer's instructions. Plates were analyzed on an ABI PRISM 7500 apparatus (Applied Biosystems, Foster City, CA, USA). The relative levels of mRNA were calculated using the ΔΔCt method.

### Western blot analysis

The cells were washed three times with ice-cold PBS and lyzed in Cell Lysis Buffer (1×). Protein levels were determined using the Lowry method, and then the samples were mixed with Laemmli buffer and denatured at 95°C for 5 min. After standard 10% SDS-PAGE separation, proteins were transferred onto PVDF membranes at 100 V. Next, the membranes were washed for 5 min in TBS-Tween buffer (TBST) (100 mM Tris - buffered saline, 140 mM NaCl and 0.1% Tween 20, pH 7.6) and the non-specific bindings were blocked for 60 min at RT with 2% or 0.5% BSA in TBST or with 5% non-fat milk solution in TBST. Further, membranes were washed three times for 5 min in TBST and incubated with the following primary antibody: mouse monoclonal anti-phospho-Tau (Ser396) (1∶500) in TBST, overnight at RT; rabbit monoclonal anti-phospho-GSK-3β (Ser9) (1∶500) in 5% non-fat milk solution in TBST, 1 h at RT and next overnight at 4°C; rabbit monoclonal anti-GSK-3β (1∶500) in a 5% non-fat milk solution in TBST, 1 h at RT and next overnight at 4°C; mouse monoclonal anti-phospho-GSK-3β (Tyr216) (1∶250) in 0.1% BSA in TBST, 2 h at RT and next overnight at 4°C; rabbit polyclonal anti-α/β-tubulin (1∶1000) in 5% BSA in TBST, overnight at 4°C. The membranes were then washed three times (5 min) in TBST and incubated for 60 min at RT with secondary antibody (anti-rabbit or anti-mouse IgG) (1∶4000) in a 5% non-fat milk/TBST. Antibodies were detected using chemiluminescent reaction and ECL reagent (Amersham Biosciences, Bath, UK) under standard conditions. After stripping, the immunolabeling of GAPDH was performed as a loading control.

### Statistical analysis

The results were expressed as mean values ± S.E.M. Differences between the means were analyzed using a Student's *t*-test between two groups or one-way analysis of variance ANOVA with Dunnett's Multiple Comparison post-hoc test among multiple groups. Statistical significance was accepted at p<0.05. The statistical analyses were performed using GraphPad Prism version 4.0 (GraphPad Software, San Diego, CA).

## Results

**Figure 1:**
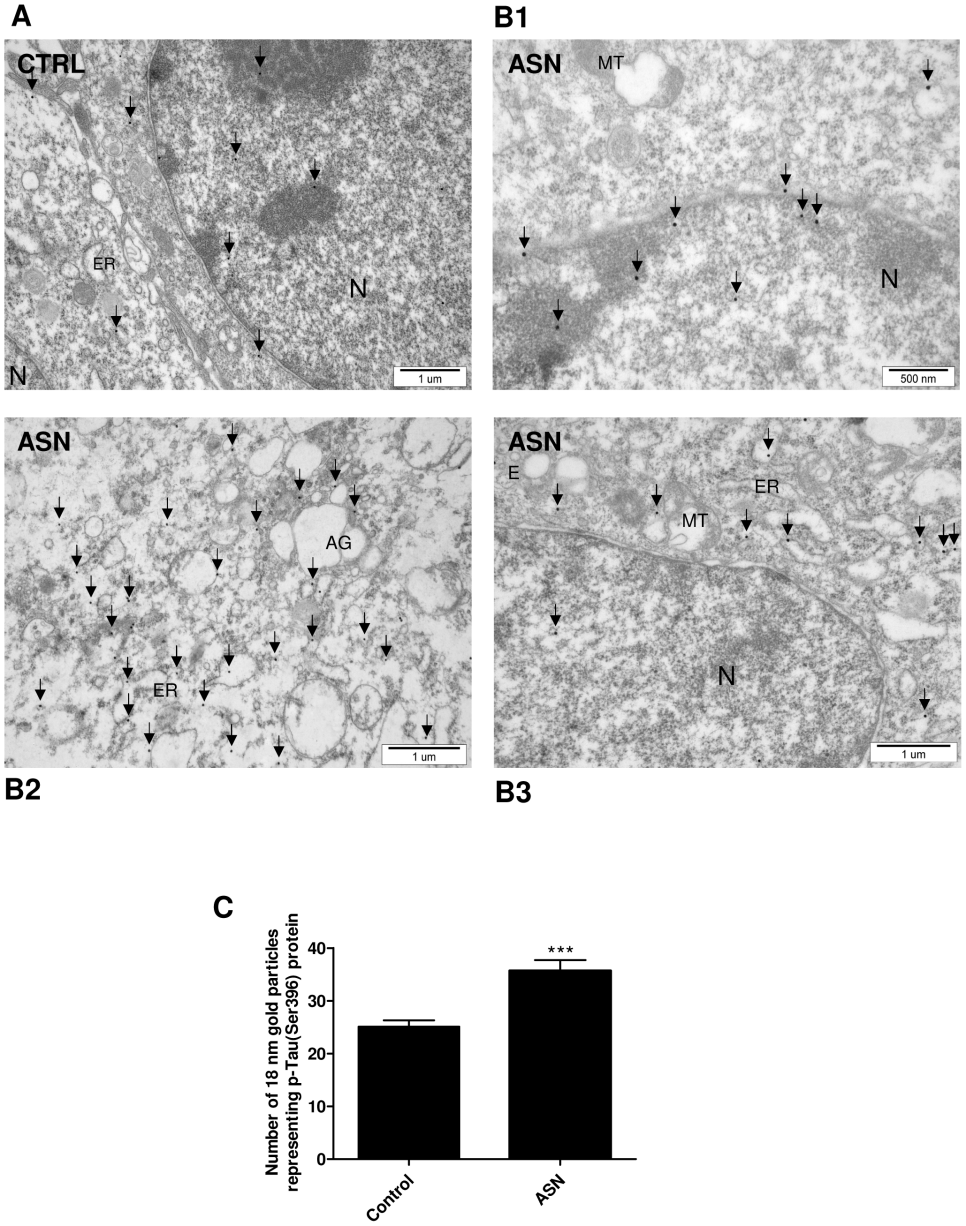
Post embedment immunostaining of p-Tau (Ser396) with 18 nm particles gold-conjugated antibody. (**A**) Control PC12 cells. (**B1–B3**) PC12 cells after 48 h treatment with 10 μM ASN. (**C**) Bar charts representing the number of gold particles representing p-Tau (Ser396) protein. Gold particles are indicated by arrows. ASN treatment resulted in significantly elevated expression of p-Tau protein in comparison to the control PC12 cells. Data represent the mean value ± S.E.M. for 3 independent experiments. *** p<0.001 *versus* control using a Student's *t*-test. Bar 1 μm. (N - nucleus, M - mitochondria, ER - endoplasmic reticulum, E - endosomes).

**Figure 2:**
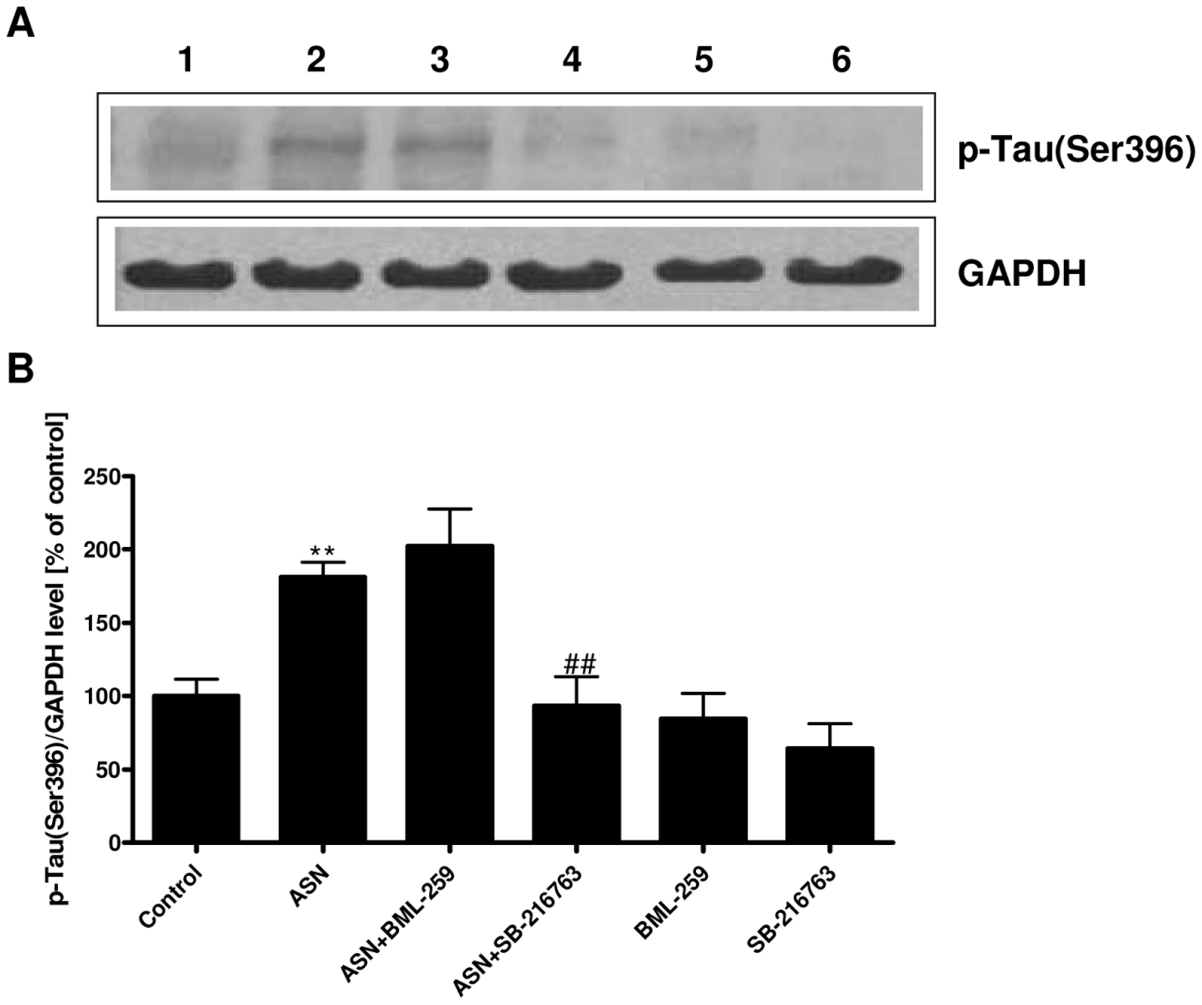
The effect of CDK5 and GSK-3β inhibitors on ASN-evoked increase in Tau phosphorylation. PC12 cells were incubated with 10 μM ASN in the presence of 10 μM inhibitors for 48 h. BML-259 and SB-216763 were used as CDK5 and GSK-3β inhibitor, respectively. The level of Tau phosphorylation at Ser396 was determined using the Western blotting method. (**A**) Immunoreactivity of p-Tau (Ser396) and GAPDH protein, which is presented as a loading control. (**B**) Densitometric analysis of p-Tau (Ser396) immunoreactivity. Results were normalized to GAPDH levels. Data represent the mean value ± S.E.M. for 3 independent experiments. ** p<0.01 *versus* control; ## p<0.01 *versus* ASN-treated cells, using a one-way ANOVA followed by Dunnett's Multiple Comparison test.

In the present study we investigated the effect of exogenous ASN on MAP-Tau protein in PC12 cells. The Tau phosphorylation status was analyzed by the TEM immunogold method. Ultrathin sections were analyzed and the mean number of gold particles representing the p-Tau protein was calculated. As presented in Figure 1, the mean number of gold particles was significantly higher in the ASN-treated cells (35.70±6.40) (Figure 1B1, 1B2, 1B3 and Figure 1C) when compared to the control cells (25.10±3.75) (p = 0.001) (Figure 1A and Figure 1C). The immunogold result was confirmed by Western blot analysis. Exogenously added ASN (10 μM) significantly increased the level of p-Tau (Ser396) by about 80% as compared to the control cells (Figure 2A and B). To evaluate the role of two major Tau-kinases: GSK-3β and CDK5 in ASN-evoked Tau phosphorylation, the inhibitors of these enzymes were used. Our results indicated that the GSK-3β inhibitor (SB-216763, 10 μM) prevented p-Tau (Ser396) formation (Figure 2A lane 4 and Figure 2B). However, the potent CDK5 inhibitor (BML-259, 10 μM) did not protect the cells against ASN-induced Tau hyperphosphorylation (Figure 2A lane 3 and Figure 2B).

**Figure 3:**
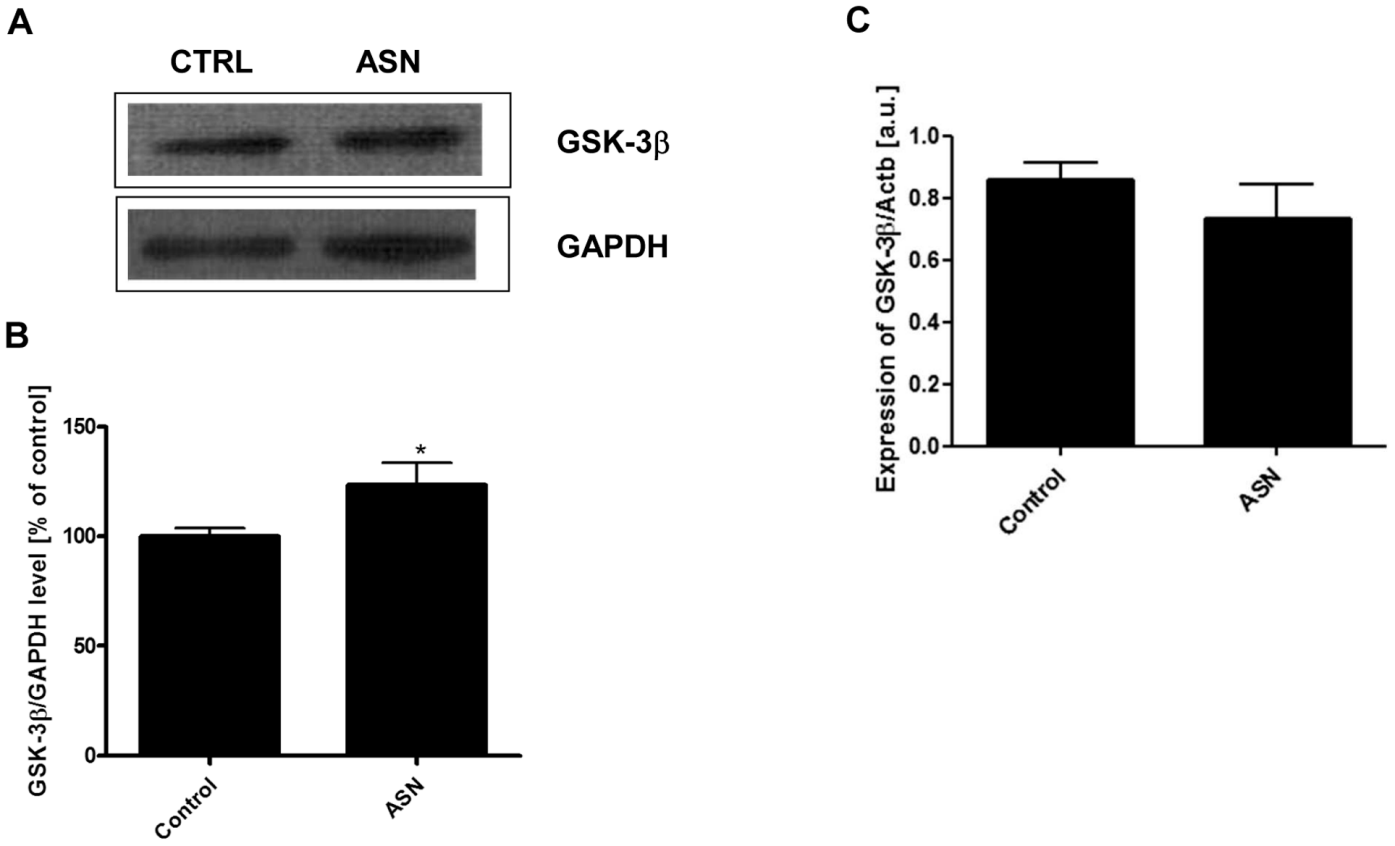
The effect of ASN on the protein level and gene expression for GSK-3β. PC12 cells were incubated in the presence of 10 μM ASN for 48 h. The total level of GSK-3β was determined using Western blot analysis. (**A**) Immunoreactivity of GSK-3β and GAPDH protein, which is presented as a loading control. (**B**) Densitometric analysis of GSK-3β immunoreactivity. Results were normalized to GAPDH levels. (**C**) The gene expression for total GSK-3β was measured with real-time PCR. Data represent the mean value ± S.E.M. for 5 independent experiments. * p<0.05 *versus* control using a Student's *t*-test.

**Figure 4:**
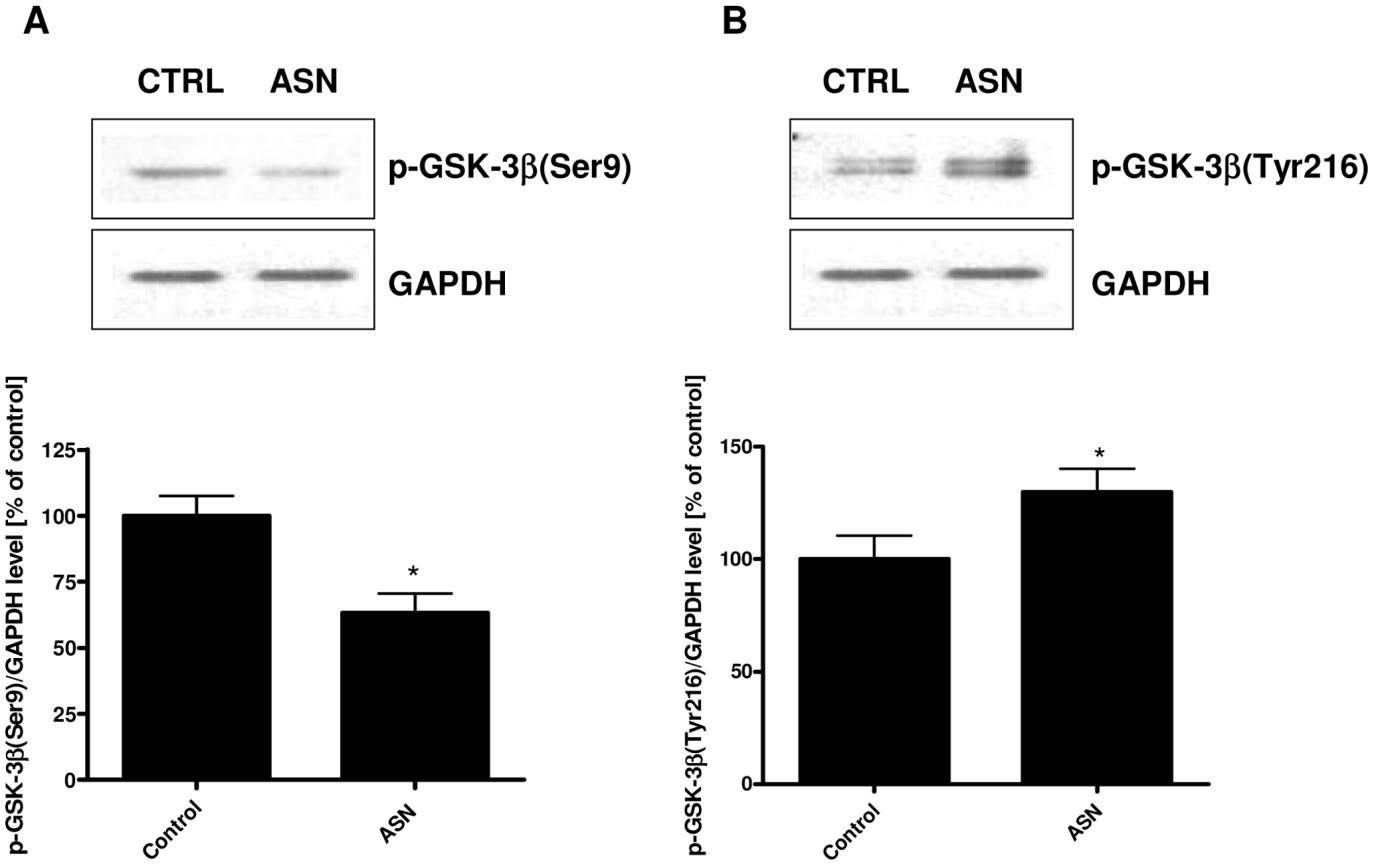
The effect of ASN on the phosphorylation state of GSK-3β. PC12 cells were incubated in the presence of 10 μM ASN for 48 h. The phosphorylation status of GSK-3β at Ser9 and Tyr216 was determined using Western blot analysis. (**A**) Immunoreactivity of p-GSK-3β (Ser9). (**B**) Immunoreactivity of p-GSK-3β (Tyr216). GAPDH was used as a loading control. Data represent the mean value ± S.E.M. for 5 independent experiments. * p<0.05 *versus* control using a Student's *t*-test.

To study the influence of ASN on GSK-3β expression, we analyzed the level of mRNA using a real-time PCR (RT-PCR) and the immunoreactivity of this enzyme by Western blot analysis. ASN treatment significantly increased GSK-3β immunoreactivity by about 24% (Figure 3A and 3B) without effect on the mRNA level (Figure 3C). GSK-3β activation was evaluated by measurement of its phosphorylation status at Ser9 and Tyr216. As presented in Figure 4A, exogenously added ASN evoked a 37% decrease in the immunoreactivity of p-GSK-3β(Ser9) with a parallel 30% increase in the p-GSK-3β(Tyr216) level (Figure 4B), thus indicating stimulation of GSK-3β activity.

**Figure 5:**
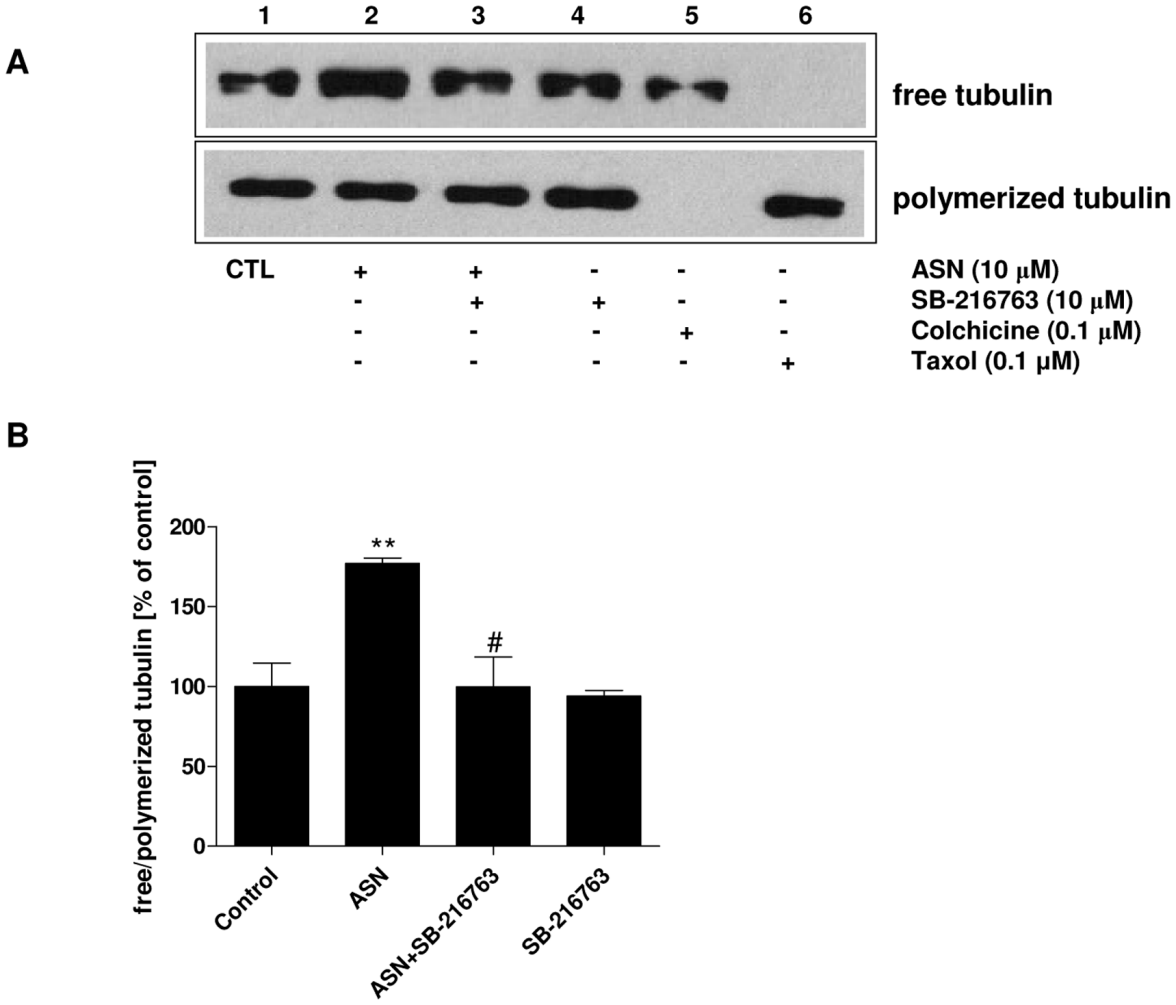
The effect of ASN on tubulin polymerization. PC12 cells were incubated with 10 μM ASN for 48 h. SB-216763, Colchicine and Taxol were used as GSK-3β inhibitor, microtubule destabilizer and microtubule stabilizer, respectively. The level of free and polymerized α/β-tubulin was determined using the Western blotting method. (**A**) Immunoreactivity of free and polymerized α/β-tubulin (**B**) Densitometric analysis of free and polymerized α/β-tubulin immunoreactivity. Microtubule instability expressed as a ratio of free to the polymerized tubulin. Data represent the mean value ± S.E.M. for 3 independent experiments. ** p<0.01 *versus* control; # p<0.01 *versus* ASN-treated cells using a one-way ANOVA followed by Dunnett's Multiple Comparison test.

To evaluate the effect of ASN on microtubule stability, we determined the amounts of free and polymerized tubulin. Our studies showed that exogenously added ASN (10 μM) significantly increased the amount of free tubulin and reduced the amount of polymerized tubulin as compared to the control group (Figure 5A lane 2 and Figure 5B). SB-216763, an inhibitor of GSK-3β kinase, prevented these ASN-induced changes in tubulin (Figure 5A lane 3 and Figure 5B). Moreover, to confirm the accuracy of this method, we used Taxol (0.1 μM), a microtubule stabilizer as a negative control and Colchicine (0.1 μM), as a positive control. Colchicine binds irreversibly to tubulin dimers and prevents addition of tubulin molecules to the fast-growing end thereby inhibiting microtubule assembly (Figure 5A lane 5). In contrast to the Colchicine, Taxol prevents disassembly of tubulin dimers thereby stabilizing microtubule and inducing bundling of microtubules (Figure 5A lane 6).

**Figure 6:**
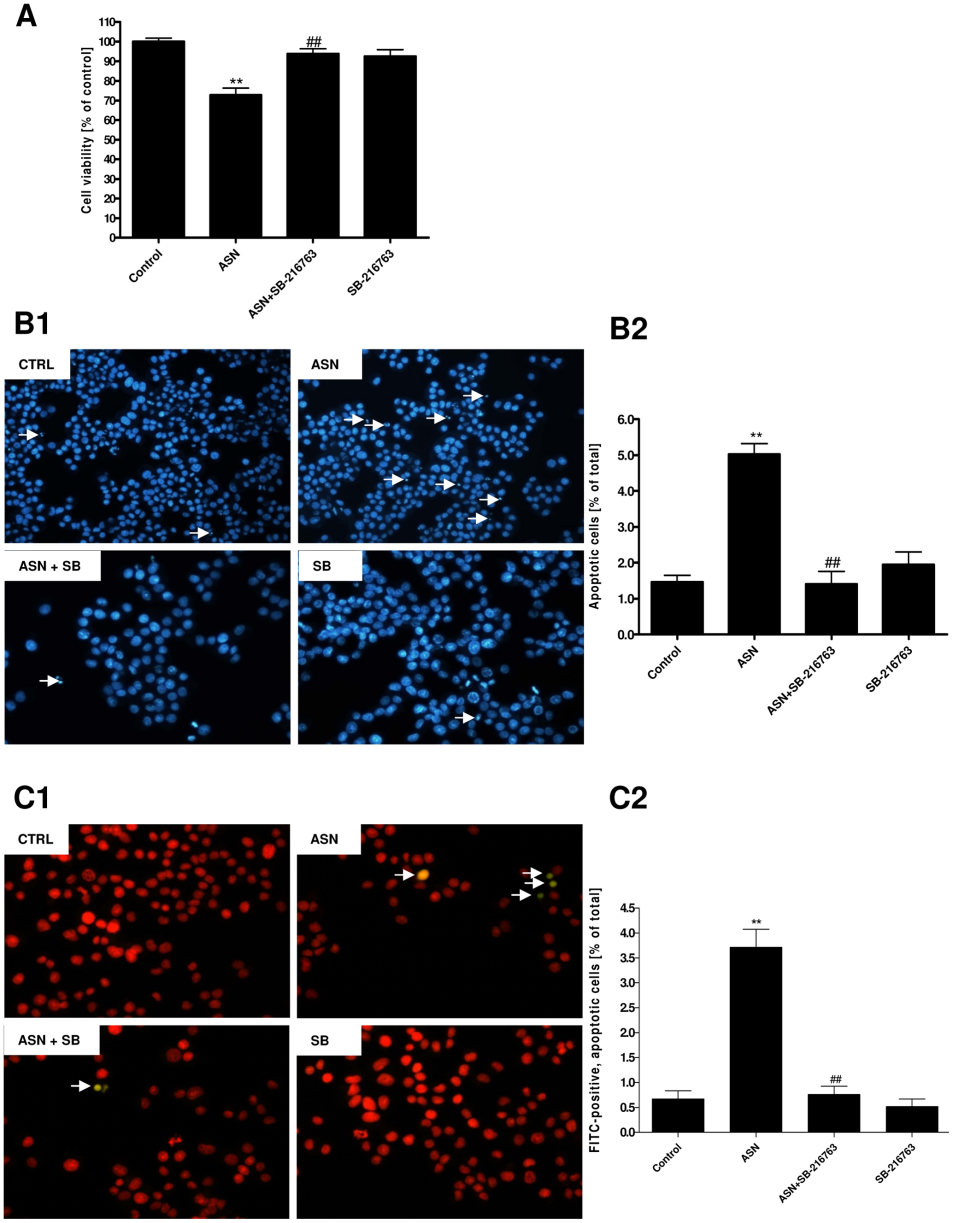
The role of Gsk-3β in apoptosis induced by ASN after 48 h treatment. (**A**) The effect of the exogenous ASN and GSK-3β inhibitor, SB-216763, on PC12 cells viability was spectrophotometrically determined using the MTT assay. ** p<0.01 *versus* control; ## p<0.01 *versus* ASN-treated cells using a one-way ANOVA followed by Dunnett's Multiple Comparison test. (**B1**) The effect of 10 μM ASN and 10 μM SB-216763 on PC12 cells apoptosis was determined *via* Hoechst 33258 fluorescent staining. The arrows indicate nuclei with typical apoptotic features. (**B2**) Apoptosis expressed as the percentage of apoptotic cells on the coverslips after 48 h treatment with 10 μM ASN. Data represent the mean value ± S.E.M. for 3 independent experiments. ** p<0.01 *versus* control; ## p<0.01 *versus* ASN-treated cells using a one-way ANOVA followed by Dunnett's Multiple Comparison test. (**C1**) The effect of 10 μM ASN and 10 μM SB-216763 on PC12 cells apoptosis was determined *via* TUNEL assay. Red colour indicates FITC-negative cells (all fixed cells stain with Propidium Iodide), green and yellow colours indicates FITC-positive cells (respectively, early and later stages of apoptotic cells characterized by specific DNA fragmentation). The arrows indicate nuclei with typical apoptotic features (FITC-positive cells). (**C2**) The number of apoptotic (FITC-stained) cells and the total number (PI-stained) of cells were counted, and apoptosis expressed as percentages of apoptotic cells (FITC-positive cells) determined within total population (PI-stained) cells on the coverslips after 48 h treatment with 10 μM ASN. Data represent the mean value ± S.E.M. for 3 independent experiments. ** p<0.01 *versus* control; ## p<0.01 *versus* ASN-treated cells using a one-way ANOVA followed by Dunnett's Multiple Comparison test.

Cell viability determined by MTT assay in the present study showed that 10 μM ASN reduced PC12 cell viability by about 27% and that SB-216763 (10 μM) prevented cell death evoked by ASN (Figure 6A).

A morphological examination of the cell nuclei stained with DNA-binding fluorochrome Hoechst 33258 showed that the PC12 cells exposed to 10 μM ASN presented typical apoptotic morphology, including condensation of chromatin and nuclear fragmentation. About 5% of the cells presented typical apoptotic morphology in the ASN group as compared to the control (*ca.* 1.5% of the total) (Figure 6B1 and 6B2). A decreased manifestation of apoptotic morphology (*ca.* 1.4% of the total) was observed when the cells were treated with exogenous ASN together with the GSK-3β inhibitor SB-216763 (10 μM) (Figure 6B1 and 6B2). The Hoechst 33258 results were confirmed by TUNEL assay. TUNEL method through reaction catalysed by exogenous TdT reveals the later step of apoptosis, DNA fragmentation, by staining terminal end of nucleic acids with FITC. About 3.7% of the cells exposed to exogenous ASN (10 μM) were FITC-positive (green and yellow colours in Figure 6C1 indicates respectively, early and later stages of apoptotic cells characterized by specific DNA fragmentation) as compared to the control group (*ca.* 0.67% of the total population cells, FITC-positive and FITC-negative cells) (Figure 6C1 and 6C2). Treatment PC12 cells with exogenous ASN together with the GSK-3β inhibitor SB-216763 (10 μM) significantly reduced level of apoptotic cells (*ca.* 0.76% of total population cells) (Figure 6C1 and 6C2).

## Discussion

A broad range of neurodegenerative disorders, including PD and AD, is characterized by neuronal damage that may be caused by toxic proteins in oligomeric form [28], [29]. Oligomers of ASN are believed to play a critical role in the pathogenesis of PD and are implicated in other neurodegenerative disorders. However, the underlying mechanism by which ASN affects neuronal function and death remains unclear. Novel data have shown elevated tauopathy in PD and have suggested a relationship between ASN and the Tau protein, thus indicating a dualism in neurodegeneration [30]. However, there are no data concerning the role of ASN in Tau modification and the eventual consequences of ASN/Tau interplay in the cell death machinery. A growing body of evidence and our previous data have indicated that ASN acts together with other amyloidogenic peptides in neurodegenerative processes, including the Aβ and Tau proteins. We have previously shown that extracellular ASN enhanced the release of Aβ peptides from APP (amyloid precursor protein)-expressing PC12 cells and potentiated its toxicity, thus leading to nitric oxide (NO)-mediated irreversible mitochondria dysfunction and caspase-dependent programmed cell death [9]. On the basis of the existing data and in light of our own results we hypothesized that the Tau protein plays an important role in ASN-evoked cytotoxicity and in PD pathology.

In this study we have shown, for the first time that extracellularly added ASN in monomeric/oligomeric form increases Tau phosphorylation on Ser396, leads to the tubulin depolymerization and that the GSK-3β inhibitor protects cells against ASN-evoked both Tau hyperphosphorylation and MT destabilization. Moreover, we showed that ASN activated GSK-3β *via* affecting its phosphorylation status. Finally, ASN-induced apoptotic processes led to PC12 cell death, and the GSK-3β inhibitor attenuated ASN-evoked cytotoxicity and enhanced PC12 cells viability.

Hyperphosphorylation of Tau has been indicated in various models of Parkinsonism and synucleinopathies and it was suggested that ASN could initiate Tau changes [3]-[5], [10], [11], [12], [18], [19], [31], [32]. Frasier et al. demonstrated that mice overexpressing A30P ASN develop abnormally phosphorylated Tau in parallel with the accumulation of aggregated ASN [5], [10]. Association of Tau pathology with synucleinopathies was also found by Duka et al., who demonstrated the ASN-mediated hyperphosphorylation of Tau at Ser396/404 in the 1-methyl-4-phenyl-1,2,3,6-tetrahydropyridine (MPTP) model of Parkinsonism [31]. In addition, Tau pathology was observed in both the platelet-derived growth factor (PDGF)-ASN mice and patients with PD [3]. Khandelwal et al. detected an increase in Tau phosphorylation at Ser396, Ser202/Thr205 and Thr231 in ASN-injected brains [32]. Moreover, *in vitro* incubation of Tau and ASN synergistically promoted the fibrillization of both proteins [19].

Since the two major protein kinases, GSK-3β and CDK5, are involved in Tau phosphorylation [4], [12], [20], we determined the contribution of these enzymes in ASN-induced Tau phosphorylation. Our results showed that only SB-216763, a GSK-3β inhibitor, had a protective effect and prevented against ASN-evoked Tau hyperphosphorylation without the effect of the CDK5 inhibitor. Thus, these results indicate the significant role of GSK-3β in ASN-induced Tau phosphorylation. The question arises as to what kind of mechanism is responsible for ASN-induced GSK-3β activation.

GSK-3β activity is regulated by serine (inhibitory) and tyrosine (stimulatory) phosphorylation, by its subcellular localization, by the formation of protein complexes containing GSK-3β and by the phosphorylation state of GSK-3β substrates *via* other kinases [33]. The activity of GSK-3β can be reduced by phosphorylation of Ser9, while optimal activation occurs through phosphorylation at Tyr216 [4], [11], [33]. Therefore, we examined the effect of exogenous ASN on the phosphorylation status of GSK-3β at these two specific sites. Our data showed that exogenously added ASN significantly decreased the phosphorylation of GSK-3β at Ser9 with a parallel increase in phosphorylation at Tyr216, thus indicating activation of this enzyme. Duka et al. showed that in several *in vitro* and *in vivo* experimental models of PD, GSK-3β was activated by phosphorylation at Tyr216 in the presence of ASN, which then led to Tau hyperphosphorylation at Ser262 and Ser396/404 [11]. In a report by Haggerty et al., in both the PDGF-ASN-overexpressing mice and PD patients, GSK-3β was phosphorylated at Tyr216 with parallel Tau hyperphosphorylation [3]. Kawakami et al. showed the stimulatory effect of ASN on the autophosphorylation of GSK-3β (although many authors support the idea that Tyr phosphorylation is regulated, there is also evidences that the Tyr phosphorylation of GSK-3 might be an autophosphorylation event catalysed by GSK-3 itself [34]) leading to its activation and GSK-3β-mediated hyperphosphorylation of Tau [18]. The effect of ASN on GSK-3β and the increase in Tau phosphorylation may explain the protective effect of GSK-3β inhibitors on PC12 cells viability. However, the precise mechanism by which exogenous ASN contributes to the activation of GSK-3β and, in consequence, to GSK-3β-dependent Tau hyperphosphorylation remains to be elucidated. Our data have suggested that ASN affected GSK-3β activity by modulating a ratio of phosphorylated and non-phosphorylated form of protein. From the literature data it follows that are several theories which attempt to explain the mechanism, by which ASN could affect on GSK-3β activation. One of them assumes that ASN translocates into the cells and forms protein complexes with GSK-3β, which then leads to changes in protein folding and structure [11]. ASN may initiate the activation of GSK-3β by recruiting this enzyme, possibly by direct protein-protein interaction, which may in turn promote conformational changes in GSK-3β, thus resulting in its autophosphorylation [3], [4]. It is also assumed that ASN functions as a connecting mediator for Tau and GSK-3β and forms a heterotrimeric complex. It was suggested that NAC domain and an acidic region of ASN are responsible for stimulation of GSK-3β-mediated Tau phosphorylation [18]. Our data demonstrate an increase in the total level of GSK-3β with a lack of changes in gene expression for this enzyme following treatment of exogenous ASN. These data may indicate either an increase in protein translation or the inhibition of processes leading to its degradation, thus causing the consequent increase in protein stability and level. However, the mechanism of GSK-3β activation requires further study and remains to be elucidated.

It is well documented that Tau is cytoskeleton protein and its binding to MT is dependent on its phosphorylation state. Highly phosphorylated Tau is less able to bind and assemble microtubules [17]–[19]. In our study, we demonstrated that ASN-induced microtubule destabilization (tubulin depolymerization) is caused by probable reduction in the binding capacity of Tau to MT(s), which is a result of Tau phosphorylation *via* GSK-3β activation. This suggests that GSK-3β has an important role in ASN-evoked MT destabilization. Disruption of tubulin polymerization into microtubules was found by Chen et al., who demonstrated the inhibition of tubulin polymerization in a dopaminergic cell line inducted by oligomeric ASN [35]. Also Lee et al. demonstrated the disruption of the microtubule network, impairment of MT-dependent trafficking and neuritic degeneration in SH-SY5Y cells overexpressing ASN [36]. Zhou et al. detected that ASN inhibited microtubule formation in the cultured cells, with a length-dependent phenomenon [37]. ASN aggregation, which occurs in PD, DLB and normal aging, has been reported to inhibit tubulin polymerization [38].

Since microtubules are involved in cytoskeletal formation, in the regulation of mitotic apparatus, in the transport of intracellular organelles such as mitochondria, in axonal transport and normal neuronal functions; disruption of microtubules (MT destabilization) can induce cell cycle arrest in M phase, formation of abnormal mitotic spindles, inhibition intracellular transport and final triggering of signals for apoptosis [15], [37], [39], [40]. In this study we have shown that extracellularly added ASN induced apoptotic processes leading to decrease of PC12 cells viability. SB-216763 protected those cells against ASN-evoked cytototoxicity. From these results, we propose that microtubule dysfunction and the associated cellular function impairment might be critical components in the mechanism of ASN-induced cell defects leading to death.

Taken together, these studies provide evidence that extracellular ASN is involved in GSK-3β-dependent Tau modulation and that its proapoptotic effect might be mediated at least in part by GSK-3β-catalysed Tau hyperphosphorylation, which leads to the tubulin depolymerization and in further consequence may impair cytoskeleton stability. GSK-3β inhibitors may be used as a potential therapeutic strategy to counteract the effects of ASN toxicity in synucleinopathies. In our study we identified the potential mechanism by which extracellular ASN may disrupt microtubule integrity in PC12 cells, which can provide insights into the early pathogenic mechanism of PD and other synucleinopathies.
